# Synchrony or asynchrony: development of facial expression recognition from childhood to adolescence based on large-scale evidence

**DOI:** 10.3389/fpsyg.2024.1379652

**Published:** 2024-04-25

**Authors:** Yihan Wang, Qian Luo, Yuanmeng Zhang, Ke Zhao

**Affiliations:** ^1^State Key Laboratory of Brain and Cognitive Science, Institute of Psychology, Chinese Academy of Sciences, Beijing, China; ^2^Department of Psychology, University of Chinese Academy of Sciences, Beijing, China; ^3^College of Letters and Science, University of California, Berkeley, Berkeley, CA, United States

**Keywords:** children, facial expression, emotion recognition, gender difference, age

## Abstract

The development of facial expression recognition ability in children is crucial for their emotional cognition and social interactions. In this study, 510 children aged between 6 and 15 participated in a two forced-choice task of facial expression recognition. The findings supported that recognition of the six basic facial expressions reached a relatively stable mature level around 8–9 years old. Additionally, model fitting results indicated that children showed the most significant improvement in recognizing expressions of disgust, closely followed by fear. Conversely, recognition of expressions of happiness and sadness showed slower improvement across different age groups. Regarding gender differences, girls exhibited a more pronounced advantage. Further model fitting revealed that boys showed more pronounced improvements in recognizing expressions of disgust, fear, and anger, while girls showed more pronounced improvements in recognizing expressions of surprise, sadness, and happiness. These clear findings suggested the synchronous developmental trajectory of facial expression recognition from childhood to adolescence, likely influenced by socialization processes and interactions related to brain maturation.

## 1 Introduction

Recognizing facial expressions is crucial for social interactions because they offer valuable insights into individuals’ internal emotions (Herba and Phillips, 2004; Watling et al., 2012; Liu et al., 2021). Via facial expressions, individuals can gather abundant information to assess others’ emotions and generate appropriate responses (Cunningham and Odom, 1986; Gao and Maurer, 2009; Johnston et al., 2011). The ability to recognize facial expressions starts to develop in infancy, as shown by research indicating that 2-month-old infants display sustained attention towards their mother’s happy expressions (Rochat et al., 2002). The developmental trajectory of children’s ability to recognize facial expressions serves as a critical measure for evaluating their emotional cognitive development (Ogren and Johnson, 2021). Facial expression recognition also plays a pivotal role in social interactions (Bayet and Nelson, 2019), thus contributing to enhancing children’s social adaptations (Ogren and Johnson, 2021).

Considering the critical role of facial expression recognition in children’s social interactions and emotional development, comprehending the developmental trajectory of facial expressions from childhood to adolescence is of paramount importance. Nevertheless, this field currently faces a shortage of research, exacerbated by relatively small sample sizes (Gao and Maurer, 2009; Rodger et al., 2015).Consequently, there are unresolved issues in facial expression recognition that require clear clarification. A primary concern is to ascertain whether there are distinct developmental stages or pivotal moments in the progression of facial expression recognition ability from childhood to adolescence. Several studies have documented notable enhancements in children’s recognition accuracy between the ages of 3 and 7, followed by a subsequent increase in recognition speed from 7 to 10 years old (Sonneville et al., 2002; Durand et al., 2007; Widen and Russell, 2008). Another study identified two primary stages in the developmental continuum of facial expression recognition: spanning from ages 5 to 12 and extending from adolescence into adulthood (Rodger et al., 2015). However, some studies have challenged this categorization, showing that 10-year-old children achieve nearly identical accuracy scores as 16-year-old adolescents in complex emotion recognition tasks (Naruse et al., 2013; Lawrence et al., 2015). A recent study additionally found that 8-year-olds surpassed 5-year-olds and performed equally to older adults (Ruffman et al., 2023). Thus, researchers in this field have not yet reached a consensus on the developmental stages of recognition ability. One plausible explanation for this inconsistency is the intricate nature of diverse emotions, which complicates the developmental process (Johnston et al., 2011; Lawrence et al., 2015; Rodger et al., 2015; Vesker et al., 2018; Louisa et al., 2019). Therefore, a larger sample size is necessary to study the synchronous characteristics of facial expression recognition development from childhood to adolescence.

Another concern in facial expression recognition development is gender differences. A meta-analysis has examined differences in decoding non-verbal emotional signals, including facial expressions, vocal prosody, postures, and gestures, between genders (Hall, 1978). The findings revealed a trend where female participants consistently outperformed their male counterparts in identifying and interpreting non-verbal cues. Furthermore, another meta-analysis supported a slight, yet robust, advantage for females in facial expression recognition from infancy through adolescence (McClure, 2000). The superiority of female participants in processing facial expressions of emotion can be explained by anatomical differences, varying rates of maturation of neurological structures responsible for emotion processing, and differences in social experiences (Bourne, 2005). While gender differences in emotion processing among children have not been consistently observed (Gross and Ballif, 1991), females, on average, show greater proficiency in understanding the emotional disposition of both genders (Kiecolt-Glaser and Newton, 2001; Rosip and Hall, 2004; Neff and Karney, 2005). However, conflicting findings regarding the influence of gender on children’s expression recognition persist. Some studies have suggested that preschool and school-age girls demonstrate slight yet consistent advantages in emotion recognition. This accelerated development in girls’ initial expression recognition ability may be attributed to their early exposure to a more expressive environment since infancy (Mancini et al., 2013; Cameron et al., 2018). Conversely, another study utilizing matching paradigms found no gender differences in emotion processing among children (Herba et al., 2006). The gender disparities in facial expression recognition between boys and girls from childhood to adolescence call for evidence from substantial samples of continuously age-staged data.

As discussed above, the developmental trajectory of facial expression recognition ability from childhood to adolescence remains unclear. Furthermore, there is a lack of consensus regarding the developmental processes of various emotions and the influence of gender differences on facial expression recognition. To tackle these challenges, the current study utilized a large sample and employed two forced-choice rapid facial expression recognition paradigms (Zhao et al., 2017, 2020), systematically assessing the developmental characteristics of children aged 6 to 15 in recognizing six basic facial expressions (happiness, disgust, anger, fear, sadness, and surprise). The two forced-choice paradigms offer distinct advantages in studying facial expressions, providing a clear and direct assessment of emotional responses. By presenting participants with two options representing different emotions, researchers were able to precisely measure and compare participants’ facial expression responses to each option. This approach minimizes ambiguity and subjectivity in facial expression assessment, establishing a structured framework for participants to make choices that are easily quantifiable and analyzable. Moreover, this method facilitates comparisons between different emotions, enabling researchers to investigate the relative strength or preference for specific emotional expressions. Overall, the two forced-choice paradigm offers a systematic and controlled approach to studying facial expressions in children, resulting in more precise and reliable measurements of emotional responses.

## 2 Methods

### 2.1 Participants

In this study, 510 children (273 boys and 237 girls) aged between 6 and 15(6 years 0 months old-15 years 12 months old, M_age_ = 10.53, SD = 2.41) were recruited and divided by age into five groups. The group aged 6–7 years comprised 89 children (M_age_ = 7.17, SD = 0.51; 49 boys). The group aged 8–9 years included 143 children (M_age_ = 9.04, SD = 0.53; 71 boys). The group aged 10–11 years consisted of 118 children (M_age_ = 10.90, SD = 0.56; 67 boys). The group aged 12–13 years comprised 104 children (M_age_ = 12.85, SD = 0.58; 56 boys). The group aged 14–15 years included 56 children (M_age_ = 14.55, SD = 0.39; 30 boys). All participants had normal vision and no known psychiatric disorders. Informed consent was obtained from each child’s legal guardian, and assent was obtained from the child. The study protocol was approved by the Institutional Review Board (IRB) at the Institute of Psychology, Chinese Academy of Sciences, and conducted in accordance with the principles outlined in the Declaration of Helsinki.

### 2.2 Materials and instruments

The present study utilized the Pictures of Facial Affect (POFA; Ekman, 1976) to assess facial expression recognition. Sixty images depicting six basic emotions (happiness, disgust, anger, fear, sadness, and surprise) were selected from the POFA dataset. To ensure comprehensive coverage, each of the six emotions was paired with every other emotion, resulting in a total of 15 different combinations: happiness-fear, happiness-anger, happiness-disgust, happiness-surprise, happiness-sadness, fear-anger, fear-disgust, fear-surprise, fear-sadness, anger-disgust, anger-surprise, anger-sadness, disgust-surprise, disgust-sadness, and surprise-sadness. The task was programmed using E-prime Version 2.0 (Psychology Software Tools, Incorporated). The stimuli were presented on a desktop computer equipped with a 60-Hz LCD monitor, with a screen resolution of 1,366 × 768 pixels.

### 2.3 Procedures

**Figure 1:**
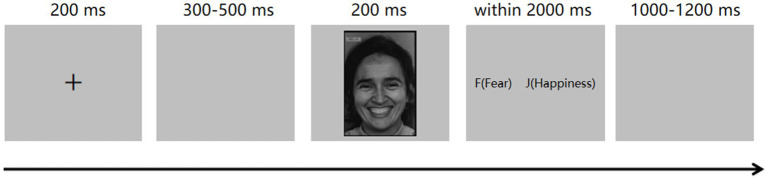
Schematic representation of the two forced-choice task.

Prior to the experiment, all participants received instructions on how to respond during the two forced-choice paradigm. They were asked to put their left index finger on the “f” key and their right index finger on the “j” key, respectively. The experimental procedure was outlined in Figure 1.

The experiment began with 4 practice trials. Each trial started with a 200 ms fixation cross, followed by a 300–500 ms blank screen. Then, a facial image was displayed for 200 ms. Participants were instructed to promptly and accurately select the correct word from a pair of words by pressing the “f” or “j” key within 2000 ms. If participants do not make a selection before the time limit expires, the word pair will vanish automatically. The interval between trials is 1,000–1,200 ms.

The test trials immediately followed the practice phase, using procedures identical to those of the practice trials. With the six basic facial expressions utilized in the paradigm, there were a total of 15 combinations. Each block presented one of these 15 combinations of facial expressions, and participants were tasked with recognizing emotions through a forced-choice task. Each facial expression category within each block consisted of 10 trials, resulting in a total of 20 trials per block. We established a total of 30 blocks, totaling 600 trials. The order of the 30 blocks was randomized for each participant. The entire experiment lasted approximately 40–50 min.

### 2.4 Statistical analysis

The data analysis in this study is structured into two main parts. The first part primarily focuses on examining the developmental patterns and gender differences related to the accuracy and reaction time associated with the six basic expressions across age groups, utilizing traditional analysis of variance (ANOVA). Subsequently, the second part employs advanced data modeling techniques to further delineate the trajectories of expression recognition performance with age, while also investigating gender differences.

In this study, a mixed-design ANOVA was conducted to analyze the effects of expression type, age group, and gender on facial expression recognition. The design consisted of a 6 (expression type: happiness, fear, anger, disgust, surprise, and sadness) × 5 (age group: 6–7 years, 8–9 years, 10–11 years, 12–13 years, and 14–15 years) × 2 (gender: female and male) factorial structure. Expression type served as the intra-group factor, while age and gender served as inter-group factors. The dependent variables were accuracy and reaction time (RT) of emotion recognition for facial expressions. Accuracy data were used as an index of facial expression emotion recognition, while reaction time data were utilized as a measure of the speed of facial expression emotion recognition. This analysis aimed to examine the developmental patterns of facial expression recognition across different age groups and explore the influence of gender on children’s expression recognition.

To characterize the increase and decrease of accuracy with respect to age for each expression, we employed General Linear Models (GLMs) across all age groups independently for each emotional expression. For each expression, we independently sampled with replacement for each group and used GLM to fit the accuracy of each expression with respect to age under each group of samples. Then, we calculated the first derivative of each fitted line (equivalent to the beta obtained by fitting a GLM with an intercept), resulting in 1000 derivative values for each emotion. Each derivative represents the rate of change in the accuracy of a specific emotion with age. Additionally, we resampled the boy and girl samples following the previous step to obtain the slope of the regression line under different genders.

## 3 Results

### 3.1 Results for analysis of variance(ANOVA) across different age groups

**Figure 2:**
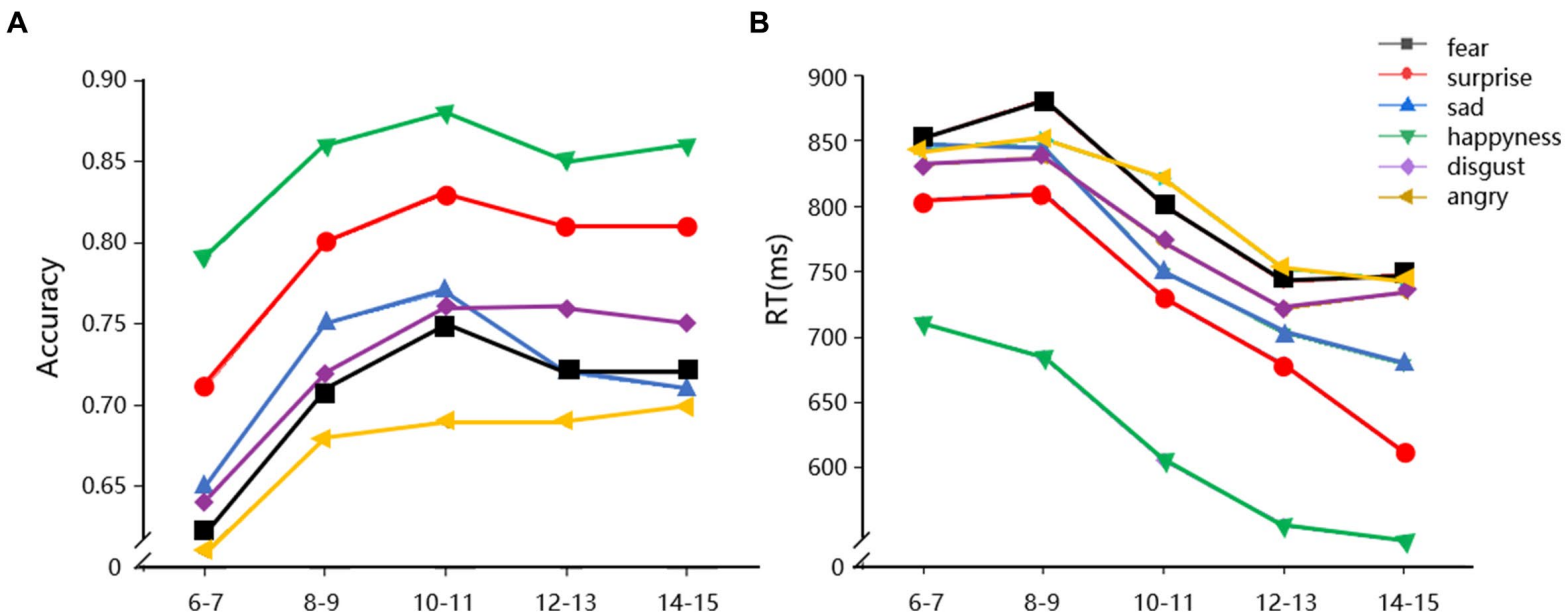
Accuracy **(A)** and reaction time **(B)** of facial expression recognition across different age groups.

The accuracy data and reaction time data of 510 children were subjected to 3-way mixed-design ANOVAs (6 emotion type×5 age group×2 gender). The analysis of accuracy revealed significant main effect for age group, *F* (4, 500) = 18.566, *p* < 0.001, $\eta_{p}^{2}$ =0.129. The accuracy of the five age groups increased, with age group 2 being the inflection point where the accuracy data began to stabilize. A significant main effect of emotion type was found (*F* (5, 2, 500) = 343.796, *p* < 0.001, $\eta_{p}^{2}$ =0.407), with the accuracy data ranking from highest to lowest for the 6 emotions as happiness, surprise, sadness, disgust, fear, and anger. There was also a significant main effect of gender [*F* (1, 500) = 9.477, *p* < 0.001, $\eta_{p}^{2}$ =0.019], with girls showing higher accuracy than boys. The interactions of age group × emotion type [*F* (20, 2, 500) = 2.804, *p* < 0.001, $\eta_{p}^{2}$ =0.022] and age group × emotion type×gender [*F* (20, 2, 500) = 2.901, *p* < 0.001, $\eta_{p}^{2}$ =0.023] were significant. However, the interaction of emotion type×gender was not statistically significant (*p* > 0.05). For boys, the interaction of age group × emotion type [*F* (4, 1, 340) = 1.818, *p* < 0.05, $\eta_{p}^{2}$ =0.026] was significant. For girls, the interaction of age group×emotion type [*F* (4, 1, 160) = 4.012, *p* < 0.001, $\eta_{p}^{2}$ =0.065] was also significant (see Figure 2A).

For reaction time, the main effect of emotion type was significant, *F* (5, 2, 500) = 241.49, *p* < 0.001, $\eta_{p}^{2}$ =0.326. The average reaction time for the 6 types of facial expression recognition is happiness, surprise, sadness, disgust, angry, and fear. The main effect of age group was also significant [*F* (4, 500) = 14.112, *p* < 0.001, $\eta_{p}^{2}$ =0.101]. The reaction time of the five age groups decreased (see Figure 2B). The interaction between emotion type and age group was significant [*F* (20, 2, 500) =3.205, *p* < 0.001, $\eta_{p}^{2}$ =0.025]. The main effect of gender, the interaction of emotion type and gender, and the interaction of emotion type, age stage, and gender were not significant.

### 3.2 General linear model regression analyses with bootstrap procedure

**Figure 3:**
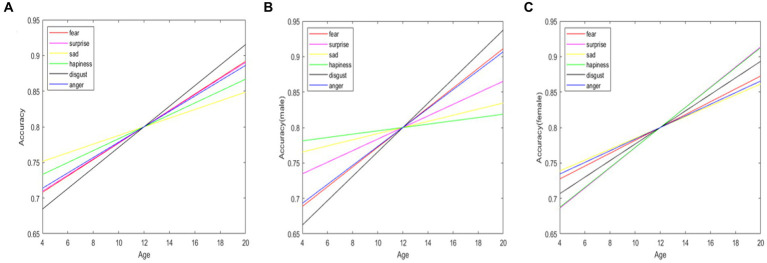
General linear model regression analysis of facial expression recognition accuracy across different age groups. **A** represents the overall population, **B** represents boys, and **C** represents girls.

Through general linear model regression analysis, it was found that the accuracy of facial expression recognition generally improves during the transition from childhood to adolescence for all facial expressions (Figure 3A). The improvement in facial expression recognition accuracy across development varies significantly for each pair of emotions, with each emotional expression exhibiting a unique trajectory across development. The expression of disgust showed the steepest improvement in recognition with age, closely followed by fear. In contrast, expressions of happiness and sadness displayed a more gradual improvement across age.

Significant differences across genders were observed in the developmental trajectory of different expressions (see Figures 3B,C). These differences can be categorized into two groups: boys showed a steeper improvement with age in recognizing expressions of disgust, fear, and anger; girls showed a steeper improvement with age in recognizing expressions of surprise, sadness, and happiness.

### 3.3 Distance matrix and multidimensional scaling analyses

**Figure 4:**
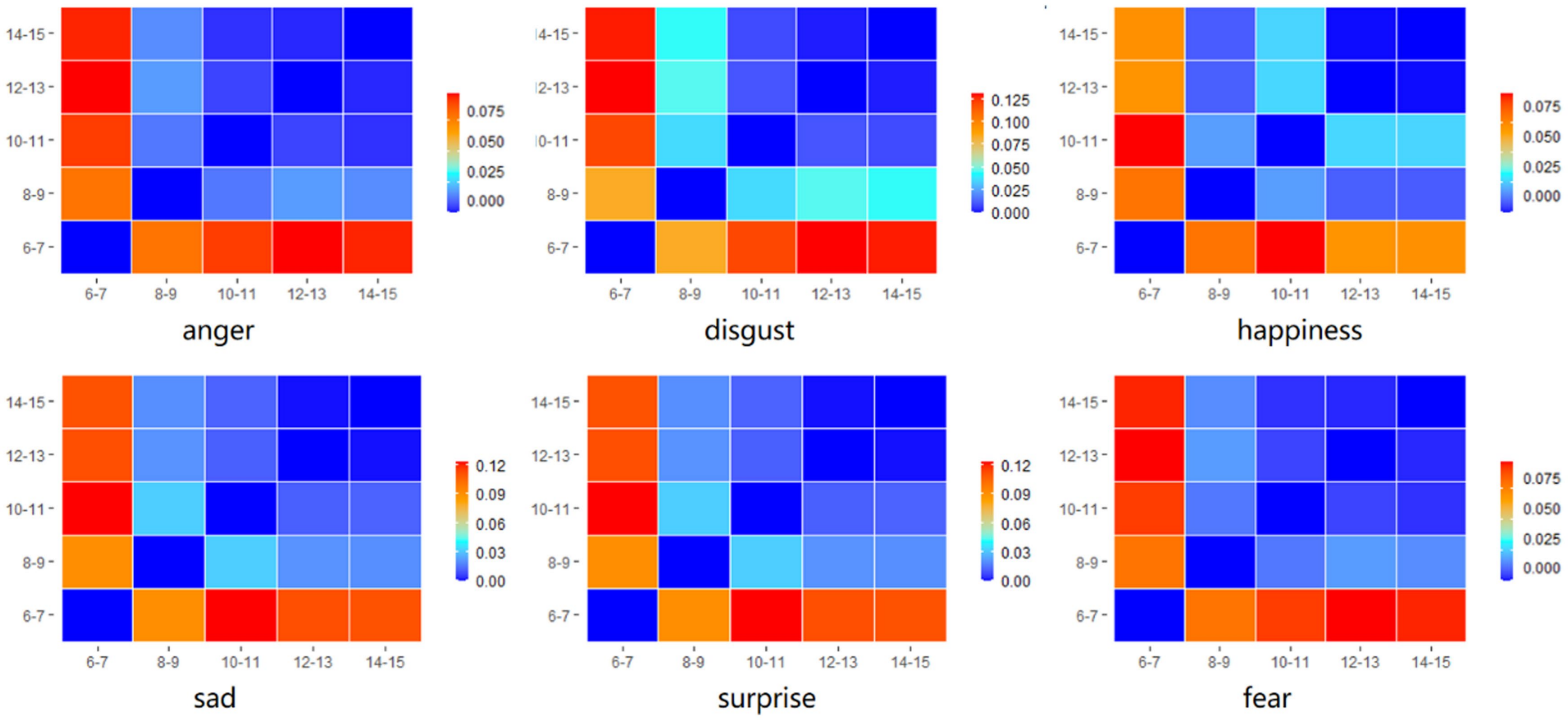
Distance matrix (i.e., matrix of pairwise difference values) of all age groups for for 6 basic facial expression.

To further investigate the relationship between emotion recognition and age, we divided the sample into five groups based on age ranges (6–7, 8–9, 10–11, 12–13, 14–15 years). Subsequently, we calculated the average accuracy for all expressions across the age groups, resulting in six values. Next, we computed the distances between each pair of age groups. Each value within this matrix indicates the distance between two age groups for a specific emotion (see Figure 4).

**Figure 5:**
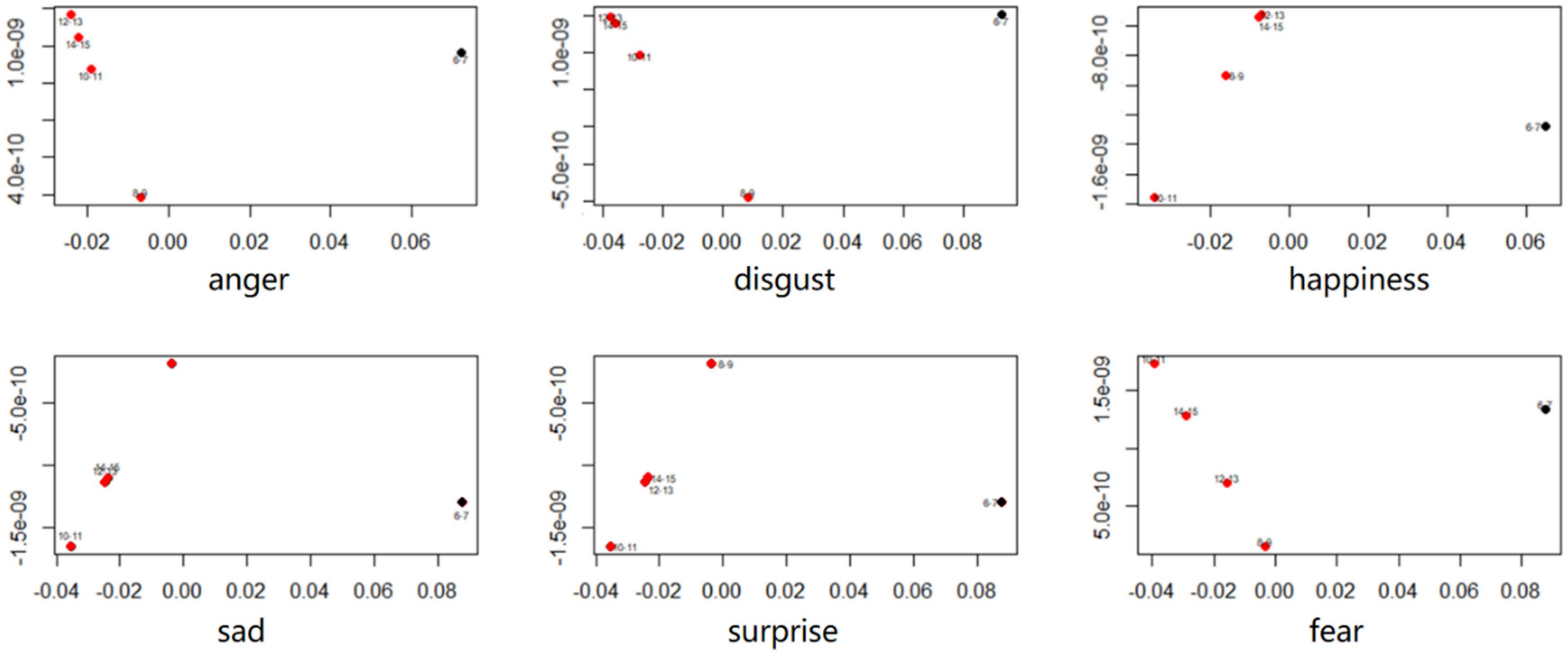
The multidimensional scaling analysis results for six basic facial expressions.

To discern the age groups that demonstrate the closest similarity, we conducted a multidimensional scaling analysis. This analysis allowed us to visualize the similarity between age groups based on their response patterns. Age groups that were placed close together on the multidimensional scaling plot indicated similar response patterns. The results of the multidimensional scaling analysis demonstrated the age groups across development that are the most similar in overall mean recognition accuracy scores (see Figure 5). Except for the 6–7 age group, the distances of age groups 8–15 are clustered together. This clustering indicates similar overall patterns and suggests that there are two main phases in the development of facial emotional expression recognition.

## 4 Discussion

This study, for the first time, explores the developmental trajectory of children’s facial expression recognition performance using a two forced-choice paradigm in a large number of children across a wide age range. There are two main findings. Firstly, the recognition performance of the six basic expressions improves with age, and around the age of 8, the recognition performance of the six basic expressions tends to stabilize. Secondly, girls outperform boys in facial expression recognition. However, the rate of development may differ between genders. These differences can be categorized into two groups: boys showed a steeper improvement with age in recognizing expressions of disgust, fear, and anger; girls showed a steeper improvement with age in recognizing expressions of surprise, sadness, and happiness.

### 4.1 The influence of age on the development of facial expression recognition

Our study demonstrated that age plays a significant role in children’s facial expression recognition. The accuracy in recognizing emotions such as anger, sadness, surprise, happiness, fear, and disgust stabilizes between the ages of 8 and 15 years. We have found that there is a continuous improvement in the proficiency of recognizing facial expressions among children transitioning into adolescence, which is consistent with previous studies (Bandura and Menlove, 1968; Gosselin et al., 1995; Vicari et al., 2000; Naruse et al., 2013; Lawrence et al., 2015; Ruffman et al., 2023). However, what sets our research apart is that we have discovered a stable age inflection point in individuals’ ability to recognize different facial expressions. Essentially, this inflection point represents a crucial moment in the process of recognizing diverse expressions.

This study provides strong evidence to clarify the key age stages of children’s facial expression recognition. From a developmental perspective, our results are inconsistent with the findings of Rodger et al. (2015) regarding the two stages of facial expression recognition development from ages 5 to 12 and from ages 13 to adulthood. Our study provides robust evidence to suggest that the recognition of facial expressions in children is closely linked to specific age stages, with a notable shift occurring around the age of 8. The primary factor contributing to the disparity between our findings and those of previous studies lies in the utilization of a significantly large sample size and a highly continuous age range in our investigation. Furthermore, it is plausible that variations in facial expression recognition paradigms may contribute to the observed discrepancies. Another aspect to consider is that the facial expression recognition paradigm we utilized involved relatively short presentation for the facial expressions, emphasizing the attributes of rapid facial expression recognition.

According to sociological theories, children’s social environment undergoes significant changes in their junior year of primary school. During this period, children begin to establish school bonding with classmates and teachers through socialization (Catalano et al., 2004). Faces are recognized as a primary tool for social communication with peers (see Jack and Schyns, 2015 for a review). Hence, during the process of bonding and communication, children could be trained to better read the facial expressions of people in the social environment, leading to rapid development in expression recognition ability (Naruse et al., 2013). It is therefore reasonable to suggest that the inflection points of children’s emotion recognition could be detected around the age of 8 years old.

In addition to social reasons, continued neurological development also explains the pattern. For example, although there may not be a one-to-one relationship between a certain brain region and specific emotion recognition, the ongoing development of the medial prefrontal cortex (MPFC) as a general region for emotion processing and the anterior cingulate cortex (ACC) as the attention regulator for emotional stimuli (see Phan et al., 2002 for a review) throughout childhood and adolescence could explain the improvement over time.

Additionally, the reaction time for children’s recognition decreases from 10 to 15 years of age. It should be noted that the inflection point in the performance of facial expression recognition does not completely coincide with the inflection point in reaction time. This finding provides further evidence to support the notion that the development of facial expression recognition and cognitive development follow distinct trajectories. Essentially, the advancement of facial expression recognition does not occur solely as a consequence of alterations in cognitive development. It also suggests that the emergence of the inflection point in children’s facial expression recognition may be influenced by the process of socialization. Due to the utilization of emotion labeling or recognition tasks in the current study, as opposed to Rodger’s study which focused on assessing perceptual thresholds, it is plausible that the observed age-related improvements in accuracy were associated with the efficiency of recognition and/or labeling processes, rather than any perceptual developments.

### 4.2 The gender difference on the development of facial expression recognition

Our findings showed that the accuracy of children’s facial expression recognition is affected by gender. In general, females are found to exhibit more acute abilities at decoding and processing discrete facial expressions (Larkin et al., 2002; Hall and Matsumoto, 2004; Passarelli et al., 2018). Additionally, girls also often obtain higher accuracy than boys (McClure, 2000). Previous studies have shown that gender poses a significant effect on the accuracy of recognizing expressions of surprise, and on average, girls exhibit more accurate recognition for expressions of anger and disgust than boys (Montirosso et al., 2010). Other studies also affirmed this gender impact on the accuracy of recognizing expressions of disgust in children aged 8–11 years (Mancini et al., 2013). Lawrence et al’s. (2015) research demonstrated that there is a female advantage in facial expression recognition, with girls exhibiting higher accuracy than boys in all ages between 6–16 years old.

Gender differences in the inflection points of recognition accuracy for expressions of surprise, happiness, and anger demonstrated a gender advantage by participants. The neural regions involved in the facial expression processing of males and females utilize different activation modes. Males and females exhibit unique activation modes in their neural regions involved in emotional facial expression processing such as the amygdala and prefrontal cortex (Mancini et al., 2013; Lawrence et al., 2015; Arriaga and Aguiar, 2019). As such, the two genders may rely on different mental processes to recognize facial expressions. However, it is worth mentioning that this gender difference decreases with age. The female’s overall advantage in facial expression recognition found by the present study is consistent with reports based on unbiased hit rates by Sasson et al. (2010), who employed static stimuli with two intensity levels. These results reveal that females are better at reading emotional facial expressions than males regardless of the degree of visual cues displayed on the face. The female’s judgment of expressions, independent of the degree of facial muscle activation, suggests that the mechanisms for reading emotions are generally better in females than in males. The females’ gender advantage in facial expression recognition (accuracy and speed) appears to be more robust when using stimuli of higher ecological validity, or stimuli incorporating a wide range of emotional variations displayed dynamically. The current study highlights the importance of employing stimuli of higher ecological validity in future research.

From an evolutionary perspective, the ability of females to more quickly and accurately recognize emotions could be associated with females’ roles as caretakers for children and families. As proposed by the primary caretaker hypothesis (Babchuk et al., 1985), fast and automatic processing of facial expression might involve innate, evolutionary mechanisms to effectively tend to offspring. Being able to instantly identify others’ emotions could allow females to make appropriate responses to the needs of others. For example, after recognizing sadness in another person, females could reciprocate with comforting behaviors, which is important for maintaining their social bonding and nurturing roles.

Conversely, females’ advantage could be acquired as part of the unique emotional experiences and expectations of their gender in socialization. According to the biosocial model (Money and Ehrhardt, 1972), once a human is born, their biological sex determines their social labeling. This labeling leads to differentiated treatments for boys and girls. Generally, females are encouraged to display and recognize emotions while males are asked to suppress them (Buck, 1977). Thus, females are more likely to possess an advantage in exposure to facial expressions compared to males. Derived from the reports by Calvo et al. (2014), this exposure advantage may lead to familiarization that facilitates the identification of facial emotional expressions. Calvo et al. (2014) proved that individuals are better at recognizing the emotions that they encounter the most frequently in social interactions simply due to familiarity. It is therefore possible that females predominantly develop better emotion-processing abilities due to more exposure to emotional displays. Social influence coupled with biological factors can result in females being more well-versed in facial expression recognition. Socialization practices and display rules may make it easier for girls to display emotion expressions. It would thus be fascinating to further investigate how gender and socialization relate to emotion recognition, such as whether gender-typical female identification and socialization are associated with better recognition and vice versa.

### 4.3 The influence of emotion type on facial expression recognition

The current study reveals that the developmental patterns for different emotions are not uniform. We found that children’s recognition of happiness has the highest accuracy, with significant differences from the other five basic expressions. This positivity bias in children has been confirmed by numerous studies (Kestenbaum and Nelson, 1992; Boyatzis et al., 1993; Lenti et al., 1999; Garcia and Tully, 2020), and it may rest in the natural facial structure it induces. Children, like adults, interpret facial expressions primarily based on basic facial features. As such, facial expressions with high similarities are easily confused, resulting in low accuracy. Compared with changes in the facial structure of negative emotions (sadness, anger, fear, disgust), the facial feature for happiness is clear and distinct, hence the happy expression could be easily recognized (Mancini et al., 2013; Sou and Xu, 2019).

On the other hand, the recognition of negative emotions, especially anger, is rather disadvantaged. Our results show that the accuracy for recognizing angry expressions is the lowest. Both anger and disgust communicate the social information of condemnation (Rot et al., 2022), which potentially explains why they are easily confused, especially why it is difficult for children to distinguish one from the other (Widen and Russell, 2010a,b; Naruse et al., 2013). An alternative explanation to the disadvantaged recognition of anger is that anger is similar to other emotions to some extent, for example, sadness (Ekman and Friesen, 1978). However, findings in other studies were inconsistent with this. One study proposed that anger, like happiness, is one of the most easily recognized emotions (Mancini et al., 2013), while Garcia and Tully (2020) stated that anger was identified more accurately than sadness, but less accurately than happiness by children aged 7–10 years old.

In the case of fear recognition, we found that the accuracy of recognizing fear was the second lowest. The difficulty in identifying fear is supported by previous studies (Matsumoto and Hwang, 2011; Mancini et al., 2013). However, this result is hardly consistent with the hypothesis of psychological evolution, which states that recognizing expressions is adaptive and allows individuals to avoid dangers in the environment. The ability to recognize fear is particularly beneficial to avoid potential threats so that individuals can better plan for their next move (such as fight or flight).

### 4.4 Limitations and direction for future studies

Although the main hypotheses are supported and the findings are mostly in line with previous studies, the current study still possesses several limitations that could be addressed in future research.

The first concerns ecological validity, as mentioned before. Only static images were employed in the current study. To improve the representation of real life, it is therefore suggested to use animated images or recorded video clips of models at different intensities in future studies. This would give participants a more vivid experience and allow for a more comprehensive evaluation of gender differences in facial expression recognition ability across different age groups.

Regarding the second limitation, participants completed a forced-choice task in this study, which is highly dependent on their verbal and visual abilities. However, for children, these two abilities are not fully developed, imposing unfair disadvantages and potentially confounding the outcomes of our study. In future studies, we recommend a combination of measurements suitable for different age groups, such as discrimination paradigms and free labeling tasks, for a more accurate assessment of facial expression recognition ability.

## 5 Conclusion

In summary, our study findings indicate several key points regarding facial expression recognition in children. Firstly, facial expression recognition accuracy improves during childhood and stabilizes between the ages of 8 and 15, showing synchronous developmental patterns across various expressions. Secondly, children exhibit a decreasing trend in reaction time for recognizing facial expressions from ages 10 to 15. Thirdly, gender influences the accuracy of facial expression recognition in children, with girls demonstrating higher accuracy compared to boys.

## Data availability statement

The original contributions presented in the study are included in the article/supplementary material, further inquiries can be directed to the corresponding author.

## Ethics statement

The studies involving humans were approved by Institute of Psychology, Chinese Academy of Sciences. The studies were conducted in accordance with the local legislation and institutional requirements. Written informed consent for participation in this study was provided by the participants' legal guardians/next of kin. Written informed consent was obtained from the individual(s) for the publication of any potentially identifiable images or data included in this article.

## Author contributions

YW: Data curation, Formal analysis, Investigation, Methodology, Writing – original draft. QL: Data curation, Formal analysis, Writing – original draft, Writing – review & editing. YZ: Investigation, Validation, Writing – original draft, Writing – review & editing. KZ: Conceptualization, Formal analysis, Funding acquisition, Investigation, Methodology, Project administration, Resources, Writing – original draft, Writing – review & editing.

## References

[ref1] Arriaga P. Aguiar C. (2019). Gender differences in aggression: the role of displaying facial emotional cues in a competitive situation. Scand. J. Psychol. 60, 421–429. doi: 10.1111/sjop.12568, PMID: 31378010

[ref2] Babchuk W. A. Hames R. B. Thompson R. A. (1985). Sex differences in the recognition of infant facial expressions of emotion: the primary caretaker hypothesis. Ethol. Sociobiol. 6, 89–101. doi: 10.1016/0162-3095(85)90002-0

[ref3] Bandura A. Menlove F. L. (1968). Factors determining vicarious extinction of avoidance behavior through symbolic modeling. J. Pers. Soc. Psychol. 8, 99–108. doi: 10.1037/h0025260, PMID: 5644484

[ref4] Bayet L. Nelson C. A. (2019). The perception of facial emotion in typical and atypical development. In LoBue V., Pérez-Edgar K., Buss K. A. (Eds.) Handbook Emot. Dev. Springer, 105–138. doi: 10.1007/978-3-030-17332-6_6

[ref5] Bourne V. J. (2005). Lateralised processing of positive facial emotion: sex differences in strength of hemispheric dominance. Neuropsychologia 43, 953–956. doi: 10.1016/j.neuropsychologia.2004.08.007, PMID: 15716165

[ref6] Boyatzis C. J. Chazan E. Ting C. Z. (1993). Preschool children's decoding of facial emotions. J. Genet. Psychol. 154, 375–382. doi: 10.1080/00221325.1993.10532190, PMID: 8245911

[ref7] Buck R. (1977). Nonverbal communication of affect in preschool in children: relationships with personality and skin conductance. J. Pers. Soc. Psychol. 35, 225–236. doi: 10.1037/0022-3514.35.4.225, PMID: 864589

[ref8] Calvo M. G. Gutiérrez-García A. Fernández-Martín A. Nummenmaa L. (2014). Recognition of facial expressions of emotion is related to their frequency in everyday life. J. Nonverbal Behav. 38, 549–567. doi: 10.1007/s10919-014-0191-3

[ref9] Cameron D. Millings A. Fernando S. Collins E. C. Moore R. Sharkey A.. (2018). The effects of robot facial emotional expressions and gender on child–robot interaction in a field study. Connect. Sci. 30, 343–361. doi: 10.1080/09540091.2018.1454889

[ref10] Catalano R. F. Haggerty K. P. Oesterle S. Fleming C. B. Hawkins J. D. (2004). The importance of bonding to school for healthy development: findings from the social development research group. J. Sch. Health 74, 252–261. doi: 10.1111/j.1746-1561.2004.tb08281.x, PMID: 15493702

[ref11] Cunningham J. G. Odom R. D. (1986). Differential salience of facial features in children's perception of affective expression. Child Dev. 57, 136–142. doi: 10.2307/1130645

[ref12] Durand K. Gallay M. Seigneuric A. Robichon F. Baudouin J. Y. (2007). The development of facial expression recognition: the role of configural information. J. Exp. Child Psychol. 97, 14–27. doi: 10.1016/j.jecp.2006.12.001, PMID: 17291524

[ref13] Ekman P. (1976). Measuring facial movement. Environ. Psychol. Nonverbal Behav. 1, 56–75. doi: 10.1007/BF01115465

[ref14] Ekman P. Friesen W. V. (1978). Facial Action Coding System: A technique for the measurement of facial action. Environ. Psychol. Nonverbal Behav. doi: 10.1037/t27734-000

[ref15] Gao X. Maurer D. (2009). Influence of intensity on children’s sensitivity to happy, sad, and fearful facial expressions. J. Exp. Child Psychol. 102, 503–521. doi: 10.1016/j.jecp.2008.11.002, PMID: 19124135

[ref16] Garcia S. E. Tully E. C. (2020). Children's recognition of happy, sad, and angry facial expressions across emotive intensities. J. Exp. Child Psychol. 197:104881. doi: 10.1016/j.jecp.2020.104881, PMID: 32559635

[ref17] Gosselin P. Roberge P. Lavallée M. (1995). The development of recognition of human facial expressions of emotion. Enfance 48, 379–396. doi: 10.3406/enfan.1995.2144

[ref18] Gross A. L. Ballif B. (1991). Children's understanding of emotion from facial expressions and situations: a review. Dev. Rev. 11, 368–398. doi: 10.1016/0273-2297(91)90019-K

[ref19] Hall J. A. (1978). Gender effects in decoding nonverbal cues. Psychol. Bull. 85, 845–857. doi: 10.1037/0033-2909.85.4.845

[ref20] Hall J. A. Matsumoto D. (2004). Gender differences in judgments of multiple emotions from facial expressions. Emotion 4, 201–206. doi: 10.1037/1528-3542.4.2.201, PMID: 15222856

[ref21] Herba C. M. Landau S. Russell T. Ecker C. Phillips M. L. (2006). The development of emotion-processing in children: effects of age, emotion, and intensity. J. Child Psychol. Psychiatry 47, 1098–1106. doi: 10.1111/j.1469-7610.2006.01652.x, PMID: 17076748

[ref22] Herba C. Phillips M. (2004). Annotation: development of facial expression recognition from childhood to adolescence: behavioural and neurological perspectives. J. Child Psychol. Psychiatry 45, 1185–1198. doi: 10.1111/j.1469-7610.2004.00316.x, PMID: 15335339

[ref23] Jack R. E. Schyns P. G. (2015). The human face as a dynamic tool for social communication. Curr. Biol. 25, R621–R634. doi: 10.1016/j.cub.2015.05.052 26196493

[ref24] Johnston P. J. Kaufman J. Bajic J. Sercombe A. Michie P. T. Karayanidis F. (2011). Facial emotion and identity processing development in 5- to 15-year-old children. Front. Psychol. 2:26. doi: 10.3389/fpsyg.2011.00026 21713170 PMC3111136

[ref25] Kestenbaum R. Nelson C. A. (1992). Neural and behavioral correlates of emotion recognition in children and adults. J. Exp. Child Psychol. 54, 1–18. doi: 10.1016/0022-0965(92)90014-W 1506820

[ref26] Kiecolt-Glaser J. K. Newton T. L. (2001). Marriage and health: his and hers. Psychol. Bull. 127, 472–503. doi: 10.1037/0033-2909.127.4.472, PMID: 11439708

[ref27] Larkin K. T. Martin R. R. McClain S. E. (2002). Cynical hostility and the accuracy of decoding facial expressions of emotions. J. Behav. Med. 25, 285–292. doi: 10.1023/A:1015384812283 12055778

[ref28] Lawrence K. Campbell R. Skuse D. (2015). Age, gender and puberty influence the development of facial expression recognition. Front. Psychol. 6:761. doi: 10.3389/fpsyg.2015.00761 26136697 PMC4468868

[ref29] Lenti C. Lenti-Boero D. Giacobbe A. (1999). Decoding of emotional expressions in children and adolescents. Percept. Mot. Skills 89, 808–814. doi: 10.2466/pms.1999.89.3.808 10665012

[ref30] Liu M. Liu C. H. Zheng S. Zhao K. Fu X. (2021). Reexamining the neural network involved in perception of facial expression: a meta-analysis. Neurosci. Biobehav. Rev. 131, 179–191. doi: 10.1016/j.neubiorev.2021.09.024, PMID: 34536463

[ref31] Louisa L. Margaret C. J. Louise P. H. (2019). Effects of induced sadness mood on facial emotion perception in young and older adults. Neuropsychol. Dev. Cogn. B Aging Neuropsychol. Cogn. 26, 319–335. doi: 10.1080/13825585.2018.1438584 29447561

[ref32] Mancini G. Agnoli S. Baldaro B. Bitti P. E. R. Surcinelli P. (2013). Facial expressions of emotions: recognition accuracy and affective reactions during late childhood. J. Psychol. 147, 599–617. doi: 10.1080/00223980.2012.727891, PMID: 24199514

[ref33] Matsumoto D. Hwang H. S. (2011). Judgments of facial expressions of emotion in profile. Emotion 11, 1223–1229. doi: 10.1037/a0024356, PMID: 21942701

[ref34] McClure E. B. (2000). A meta-analytic review of sex differences in facial expression processing and their development in infants, children, and adolescents. Psychol. Bull. 126, 424–453. doi: 10.1037/0033-2909.126.3.424, PMID: 10825784

[ref35] Money J. Ehrhardt A. A. (1972). Man and woman, boy and girl: Differentiation and dimorphism of gender identity from conception to maturity. Baltimore, MD: Johns Hopkins University Press.

[ref36] Montirosso R. Peverelli M. Frigerio E. Crespi M. Brogatti R. (2010). The development of dynamic facial expression recognition at different intensities in 4- to 18-year-olds. Soc. Dev. 19, 71–92. doi: 10.1111/j.1467-9507.2008.00527.x

[ref37] Naruse S. Hashimoto T. Mori K. Tsuda Y. Takahara M. Kagami S. (2013). Developmental changes in facial expression recognition in Japanese school-age children. J. Med. Investig. 60, 114–120. doi: 10.2152/jmi.60.114, PMID: 23614919

[ref38] Neff L. A. Karney B. R. (2005). Gender differences in social support: a question of skill or responsiveness? J. Pers. Soc. Psychol. 88, 79–90. doi: 10.1037/0022-3514.88.1.79 15631576

[ref39] Ogren M. Johnson S. P. (2021). Factors facilitating early emotion understanding development: contributions to individual differences. Hum. Dev. 64, 108–118. doi: 10.1159/000511628, PMID: 34305161 PMC8301206

[ref40] Passarelli M. Masini M. Bracco F. Petrosino M. Chiorri C. (2018). Development and validation of the facial expression recognition test(FERT). Psychol. Assess. 30, 1479–1490. doi: 10.1037/pas0000595, PMID: 30024180

[ref41] Phan K. L. Wager T. Taylor S. F. Liberzon I. (2002). Functional neuroanatomy of emotion: a meta-analysis of emotion activation studies in PET and fMRI. NeuroImage 16, 331–348. doi: 10.1006/nimg.2002.1087, PMID: 12030820

[ref42] Rochat P. Striano T. Blatt L. (2002). Differential effects of happy, neutral, and sad still-faces on 2-, 4-and 6-month-old infants. Infant Child Dev. Int. J. Res. Pract. 11, 289–303. doi: 10.1002/icd.259

[ref43] Rodger H. Vizioli L. Ouyang X. Caldara R. (2015). Mapping the development of facial expression recognition. Dev. Sci. 18, 926–939. doi: 10.1111/desc.12281 25704672

[ref44] Rosip J. C. Hall J. A. (2004). Knowledge of nonverbal cues, gender, and nonverbal decoding accuracy. J. Nonverbal Behav. 28, 267–286. doi: 10.1007/s10919-004-4159-6

[ref45] Rot M. Friederici C. Krause S. C. de Jong P. J. (2022). Interpersonal responses to facial expressions of disgust, anger, and happiness in individuals with varying levels of social anxiety. PLoS One 17:e0263990. doi: 10.1371/journal.pone.0263990 35390004 PMC8989355

[ref46] Ruffman T. Kong Q. Lim H. M. Du K. Tiainen E. (2023). Recognition of facial emotions across the lifespan: 8-year-olds resemble older adults. Br. J. Dev. Psychol. 41, 128–139. doi: 10.1111/bjdp.12442, PMID: 36773033

[ref47] Sasson N. J. Pinkham A. E. Richard J. Hughett P. Gur R. E. Gur R. C. (2010). Controlling for response biases clarifies sex and age differences in facial affect recognition. J. Nonverbal Behav. 34, 207–221. doi: 10.1007/s10919-010-0092-z

[ref48] Sonneville L. M. J. Verschoor C. A. Njiokiktjien C. Veld V. O. Toorenaar N. Vranken M. (2002). Facial identity and facial emotions: speed, accuracy, and processing strategies in children and adults. J. Clin. Exp. Neuropsychol. 24, 200–213. doi: 10.1076/jcen.24.2.200.989, PMID: 11992203

[ref49] Sou K. L. Xu H. (2019). Brief facial emotion aftereffect occurs earlier for anger than happy adaptation. Vis. Res. 162, 35–42. doi: 10.1016/j.visres.2019.07.002, PMID: 31325461

[ref50] Vesker M. Bahn D. Degé F. Kauschke C. Schwarzer G. (2018). Developmental changes in the categorical processing of positive and negative facial expressions. PLoS One 13:e0201521. doi: 10.1371/journal.pone.0201521 30075000 PMC6075754

[ref51] Vicari S. Reilly J. S. Pasqualetti P. Vizzotto A. Caltagirone C. (2000). Recognition of facial expressions of emotions in school-age children: the intersection of perceptual and semantic categories. Acta Paediatr. 89, 836–845. doi: 10.1111/j.1651-2227.2000.tb00392.x 10943968

[ref52] Watling D. Workman L. Bourne V. J. (2012). Emotion lateralisation: developments throughout the lifespan. Laterality 17, 389–411. doi: 10.1080/1357650X.2012.682160 22690893

[ref53] Widen S. C. Russell J. A. (2008). Children acquire emotion categories gradually. Cogn. Dev. 23, 291–312. doi: 10.1016/j.cogdev.2008.01.002

[ref54] Widen S. C. Russell J. A. (2010a). Children’s scripts for social emotions: cause and consequences are more central than are facial expressions. Br. J. Dev. Psychol. 28, 565–581. doi: 10.1348/026151009X457550d, PMID: 20849034

[ref55] Widen S. C. Russell J. A. (2010b). The “disgust face” conveys anger to children. Emotion 10, 455–466. doi: 10.1037/a0019151 20677863

[ref56] Zhao K. Liu M. Gu J. Mo F. Fu X. Liu C. H. (2020). The preponderant role of fusiform face area for the facial expression confusion effect: an MEG study. Neuroscience 433, 42–52. doi: 10.1016/j.neuroscience.2020.03.001, PMID: 32169552

[ref57] Zhao K. Zhao J. Zhang M. Cui Q. Fu X. L. (2017). Neural responses to rapid facial expressions of fear and surprise. Front. Psychol. 8:761. doi: 10.3389/fpsyg.2017.00761, PMID: 28539909 PMC5424260

